# Methylenecyclopropylglycine and hypoglycin A intoxication in three Pére David's Deers (*Elaphurus davidianus*) with atypical myopathy

**DOI:** 10.1002/vms3.406

**Published:** 2020-12-13

**Authors:** Mandy Bochnia, Eva Ziemssen, Johannes Sander, Birgit Stief, Annette Zeyner

**Affiliations:** ^1^ Institute of Agricultural and Nutritional Sciences Martin Luther University Halle‐Wittenberg Halle (Saale) Germany; ^2^ Zoo Dresden GmbH Dresden Germany; ^3^ Screening Labor Hannover Ronnenberg Germany; ^4^ Landesuntersuchungsanstalt für das Gesundheits‐ und Veterinärwesen Dresden Germany

**Keywords:** atypical myopathy, hypoglycin A, MCPrG, methylenecyclopropylglycine, rhabdomyolysis, sycamore maple seeds

## Abstract

**Background:**

Hypoglycin A (HGA) and methylenecyclopropylglycine (MCPrG) from seeds/seedlings of Sycamore maple (SM, *Acer pseudoplatanus*) causes atypical myopathy (AM) in horses. AM was not known to occur in wild ruminants until several fatalities in milus (*Elaphurus davidianus*) following the ingestion of HGA in SM seeds. However, a role for MCPrG has not previously been evaluated.

**Objectives:**

To test the hypothesis that MCPrG is also a major factor in AM in milus, three milus (M1, M2, M3) from the Zoo Dresden (aged 7–11 years, 2 females and 1 male, in good nutritional condition) that developed AM were studied.

**Methods:**

Serum, urine and methanol extracts from the liver, kidney, rumen digesta and faeces were analysed by ultrahigh‐performance liquid chromatography‐tandem mass spectrometry for HGA, MCPrG and for conjugates of carnitine (C) and glycine (G): Methylenecyclopropylacetyl (MCPA)‐G, MCPA‐C, Methylenecyclopropylformyl (MCPF)‐G, MCPF‐C, butyryl‐C and isobutyryl‐C.

**Results:**

HGA in serum was high (M2 480 nmol/L; M3 460 nmol/L), but MCPrG was not. HGA and MCPrG were found in rumen and faeces extracts, and MCPrG was also identified in the liver. Metabolites of HGA and MCPrG were high in serum, urine and liver, but not in the rumen or faeces.

**Conclusions:**

This study shows that MCPrG is involved in the pathophysiology of AM in milus. The metabolism of MCPrG is considered to be faster because, after ingestion, the specific metabolites appear highly concentrated in the serum. The high toxin concentration in the liver suggests that a possible transfer into products for human consumption may pose a risk.

## Graphical abstract

In humans and equines, the intoxication with Methylenecyclopropylglycine (MCPrG) and Hypoglycin A (HGA) from Sapindaceae fruits or seeds is well‐known with a high fatality rate, whereas in horses the common ingestion of both toxins was firstly described in 2019. This study shows for the first time, that also in ruminants both toxins present a major hazard. A possible transfer to the food chain following the consumption of products from affected ruminants by humans should be taken into consideration.

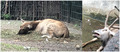

## 1 INTRODUCTION

The intake of hypoglycin A (HGA) and methylenecyclopropylglycine (MCPrG) from the seeds/seedlings of Sycamore maple (SM, *Acer pseudoplatanus)* causes atypical myopathy (AM) in pastured horses (Bochnia et al., 2019). Previous studies often used MCPG as an abbreviation for methylenecyclopropylglycine (C_6_H_9_NO_2_, 127.14 g/mol molecular weight), but the correct short form is MCPrG to clearly differentiate it (National Center for Biotechnology Information. PubChemDatabase. CID = 142776, MCPrG) and to avoid confusion with MCPG (α‐Methyl‐4‐carboxyphenylglycine, C_10_H_11_NO_4_, 209.2 g/mol molecular weight; National Center for Biotechnology Information. PubChem Database. CID = 4479247, (S)‐Mcpg). Within the scope of AM the same chemical compound, the non‐proteinogenic amino acids (AA) ‘methylenecyclopropylglycine’, was always mentioned and the solely used abbreviation for it differed from this study.

In humans, toxicity from both HGA and MCPrG was first described after the ingestion of unripened soapberry fruits (Isenberg et al., 2016), which are related to maple trees and belong to the family of Sapindaceae. It has long been recognized that ackee fruits (Scott, 1916) and litchi fruits can lead to death during harvesting, especially in children (Das et al., 2015; Gray & Fowden, 1962; Isenberg et al., 2015; John & Das, 2014; Shah & John, 2014; Shrivastava et al., 2017; Shrivastava et al., 2015). The intake of ackee and litchee fruits leads to the production of toxic metabolites and the detection of HGA (John & Das, 2014; Shah & John, 2014; Shrivastava et al., 2017; Shrivastava et al., 2015) and MCPrG (Isenberg et al., 2015; Das et al., 2015; Sander et al., 2017) in blood and urine samples from affected humans. The specific end products of the metabolism of HGA and MCPrG in serum and urine are methylenecyclopropylacetyl‐glycine and ‐carnitine (MCPA‐G and MCPA‐C, respectively) and methylenecyclopropylformyl‐glycine and ‐carnitine (MCPF‐G and MCPF‐C, respectively), which can only be detected in diseased humans (Isenberg et al., 2016) and horses (Bochnia et al., 2019). The histopathological examination of dissected animals has demonstrated the presence of a severe acute rhabdomyolysis (Żuraw et al., 2016) with extensive and finely dispersed intracellular lipid droplets in affected skeletal muscles, which confirm a lipid‐accumulation myopathy. Furthermore, highly concentrated acyl‐carnitines and acyl‐glycines are provoked by the interruption of fatty acid β‐oxidation resulting from the inhibition of acyl‐CoA‐dehydrogenases and enoyl‐CoA‐dehydratases are observed. The presence of MCPrG alone affects only enoyl‐CoA‐dehydratases during the second step of β‐oxidation that takes place within the mitochondria (Bochnia et al., 2019; Isenberg et al., 2015, 2016; Sander et al., 2019). In contrast, the presence of HGA alone mainly affects the first step of β‐oxidation or several acyl‐CoA‐dehydrogenase enzymes, which are located outside of the mitochondria. Studies have shown that the simultaneous action of both toxins intensifies the inhibiting effects during β‐oxidation (Bochnia et al., 2019; Isenberg et al., 2016).

AM was not known to occur in wild ruminants until several fatalities were identified in Pére David Deer's (*Elaphurus davidianus*), also known as milus, in association with the ingestion of HGA from SM seeds (Bunert et al., 2018). The HGA exposure in affected deers was confirmed biochemically, but interestingly, some healthy animals also had HGA in their serum and were asymptomatic. This finding, similarly to data in horses (Bochnia et al., 2018), led to the suggestion that HGA is not the only toxin in AM (Bunert et al., 2018). It supports the statement that the presence of HGA is not a predictor of AM (Bochnia et al., 2018) and that the detection of the metabolites is only associated with the outbreak of AM. Furthermore, these data strengthen the hypothesis that a second identified toxin in AM in equines (Bochnia et al., 2019) and humans (Isenberg et al., 2015, 2016), MCPrG, might also be involved in the pathophysiology of AM in milus. Given that AM in ruminants was not identified until 2018, it has been proposed that extensive microbial fermentation in rumen may decompose the toxins and thus decrease their absorption. A preliminary in vitro study indicated that a low removal of HGA resulted from incubation with the caecum content of horses, and the products of microbial degradation, if any, remain unknown (Krägeloh et al., 2017). These results go against the theory of the ruminal environment being a protective mechanism.

The objective of this study was to analyse rumen digesta, serum, urine and organ samples of affected deers for the presence of MCPrG and HGA as well as their specific metabolites. We hypothesized that, in addition to HGA, MCPrG is also involved in AM in milus.

## 2 MATERIALS AND METHODS

### 2.1 Animals

In September 2018, three milus from the Zoo Dresden (M1‐3; aged 7–11 years, 2 females, 1 male, all in a good nutritional condition) developed AM with evidence of trembling, muscle pain, salivation, dysphagia and recumbency after the ingestion of SM seeds and leaves. Since the animal keepers were not aware of the potential danger associated with SM, they fed the milus with the leaves and branches after pruning the surrounding SM trees. The course of the disease was dramatic, because all three milus developed AM symptoms within a few hours of each other and died or were shot by the Zoo's veterinarian within 2 days of symptom onset.

### 2.2 Pathology

M2 and M3 were dissected. Necropsies were performed and tissue samples from the liver, kidney, myocardium, as well as different skeletal muscles including the diaphragm, forelimb muscles and hindlimb muscles, were taken for histopathological investigation. All tissues were immersion‐fixed in 4% neutral‐buffered formalin for 24 hr, embedded in paraffin, and cut at a thickness of 4 µm. The tissue sections were subsequently stained with haematoxylin and eosin and periodic acid‐Schiff.

### 2.3 Plant sampling

Available parts of the SM tree were collected from the milus' enclosure directly after the outbreak of the disease (the vegetation period at that time: seeds and leaves) and were kept frozen (−20°C) until analysis.

### 2.4 Measurements performed in available samples

#### 2.4.1 Blood and urine samples of affected deers

Creatine kinase (CK) and lactate dehydrogenase (LDH) were analysed in blood serum (Hitachi 912; Roche Diagnostics, GmbH). Serum and urine samples from the M2 and M3 milus were analysed using ultrahigh‐performance liquid chromatography‐tandem mass spectrometry (UPLC‐MS/MS; Sander et al., 2019) for the detection of HGA, MCPrG, the conjugates of carnitine (C) and glycine (G; MCPA‐G, MCPA‐C, MCPF‐G and MCPF‐C), and the two selected acyl conjugates butyryl‐C and isobutyryl‐C. The internal standard solution contained ^13^C_2_^15^N‐MCPG, ^13^C_2_^14^N MCPF‐glycine, ^13^C_2_^14^N MCPA‐glycine. As no authentic material was available, we used d3 leucine as internal standard for HGA, d7 butyrylcarnitine for MCPF‐C and d3 octanoylcarnitine for MCPA‐C. The acyl conjugates show a strong correlation with MCPF‐C and appear mainly due to the presence of MCPF‐CoA, which is an intermediary product in the metabolism of MCPrG (Bochnia et al., 2019). Other medium‐ or long‐chain acyl conjugates were not evaluated as part of this study. Absorption may differ between ruminants and monogastric animals.

#### 2.4.2 Tissue and digesta samples

To explore the distribution of substances in the affected organisms, the involved organs/tissues (liver and kidney), rumen digesta and faeces were also sampled.

### 2.5 Analytical procedure for collected samples

A small amount of the plant material, tissue, or digesta samples (10–50 mg) were ultrasonicated. After centrifugation at 17,000 Relative Centrifugal Force (RCF) a clear supernatant was obtained. A mixture of methanolic internal standard solution (300 μl) and 250 μl of this fluid was vortexed for 20 s and again centrifuged for 10 min at 17,000 RCF. A total of 250 μl of the clear supernatant were dried in a microtiter plate at 65°C for approximately 30 min under a gentle stream of nitrogen. For the analysis of serum and urine, only 25 µl of the fluid was used with the procedure otherwise unchanged. The corresponding residues were treated with 50 μl of 3 N butanolic HCl each for 15 min at 65°C and dried again at 65°C under nitrogen. The dry material was dissolved in 70 μl of methanol/water (80:20 vol/vol) and further diluted with water in a 1:2 ratio. From this solution, 90 μl was transferred to a 384‐well microtiter plate, centrifuged at 17,000 RCF in order to sediment any particles. UPLC‐MS/MS analysis was performed on a Xevo TQ‐MS UPLC‐MS/MS system equipped with an ACQUITY UPLC BEH C18 column (1.7 μm, 2.1 mm × 50 mm; Waters) as described previously (Sander et al., 2019).

Linearity for the determination of HGA was demonstrated for a range from 10 nmol/L to 10,000 nmol/L of urine or serum (Sander et al., 2016). For MCPrG, linearity was proven in the range from 0.5 nmol/L to 500 nmol/L (Sander et al., 2019). In addition, linearity was tested and proven for the glycine and carnitine conjugates of methylene cyclopropyl acetic acid and formic acid over a range of 5–5,000 nmol/L (Bochnia et al., 2019). The intra assay coefficient of variation was below 10% for concentrations starting from 50 nmol/L for MCPF‐G, MCPA‐G and HGA in spiked serum or urine. For MCPF‐C and MCPA‐C the corresponding dose was 100 nmol/L (Bochnia et al., 2019; Sander et al., 2019).

## 3 RESULTS

### 3.1 Animals

**FIGURE 1:**
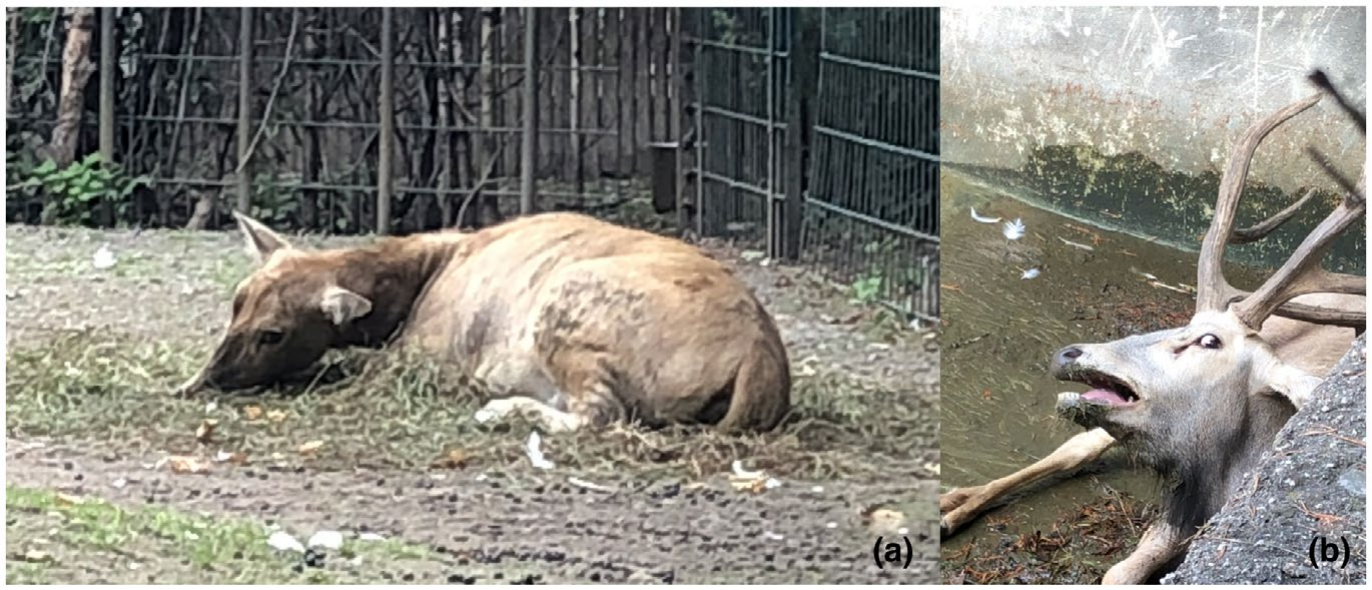
Final stage of atypical myopathy (a) of the milu with excitations (b)

**FIGURE 2:**
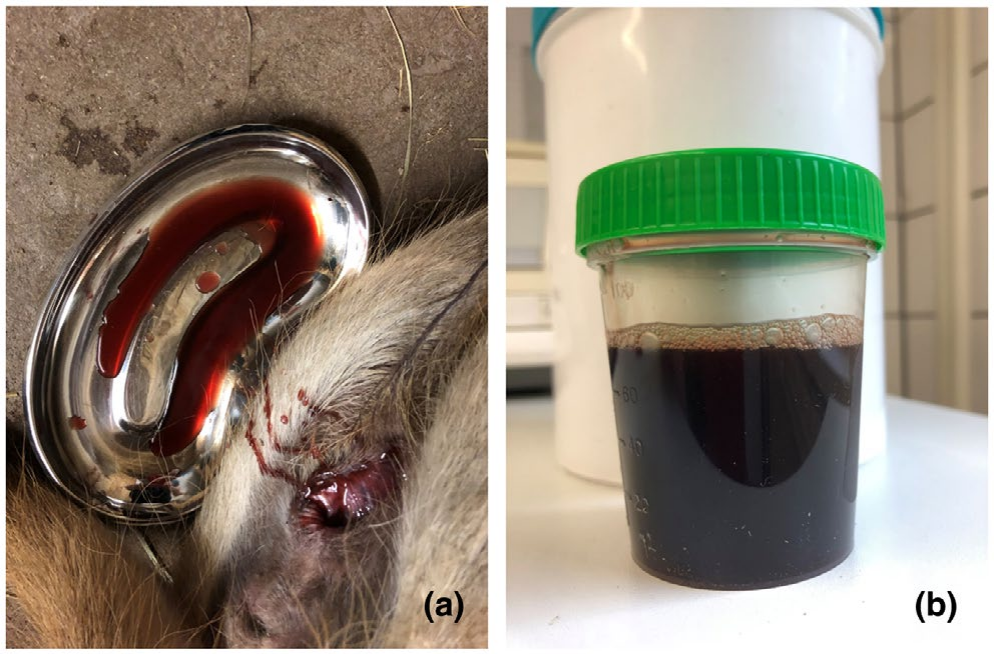
Myoglobinuria from the bladder after death (a) and the dark‐brown urine which was sampled (b)

Figures 1 and 2 show the typical symptoms of the diseased milus. In Figure 1, the final stage of the AM illness is illustrated, namely recumbency and weakness (A) as well as hyperextensions (B). Figure 2 demonstrates the run off, drop by drop, from dark‐brown urine from the bladder after death (myoglobinuria) (A), as well as the sampled urine (B).

### 3.2 Plant samples

HGA concentrations varied from 19.1 µg/g in leaves to 46.1 µg/g in the seeds. The concentration of MCPrG ranged from 0.1 µg/g in leaves and to 42.9 µg/g in seeds.

### 3.3 Pathology

**FIGURE 3:**
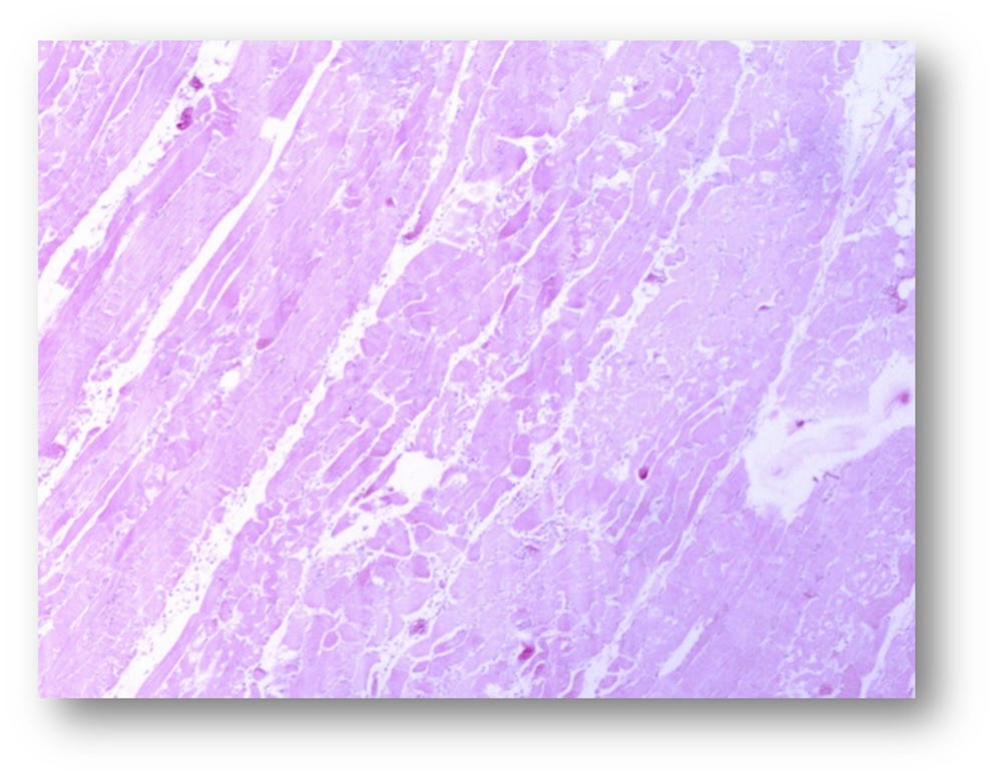
Pathohistological micrograph of the skeletal muscle from animal 2 (magnification ×2.5) after haematoxylin and eosin staining

**FIGURE 4:**
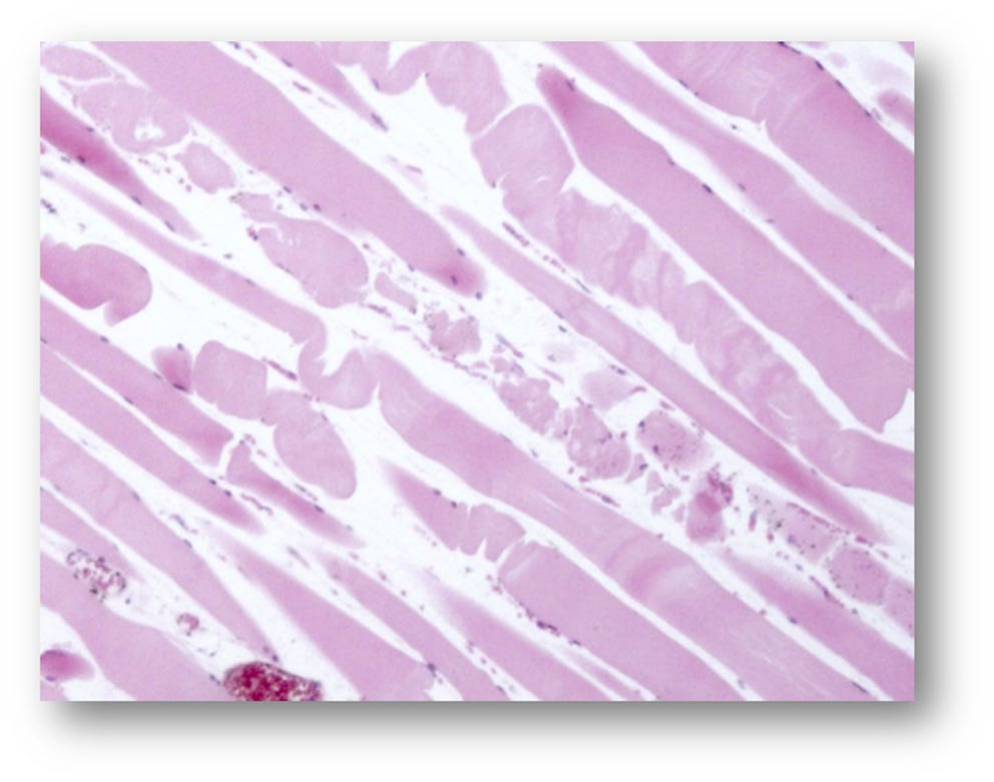
Pathohistological micrograph of the skeletal muscle from animal 1 (magnification ×10) after haematoxylin and eosin staining

**FIGURE 5:**
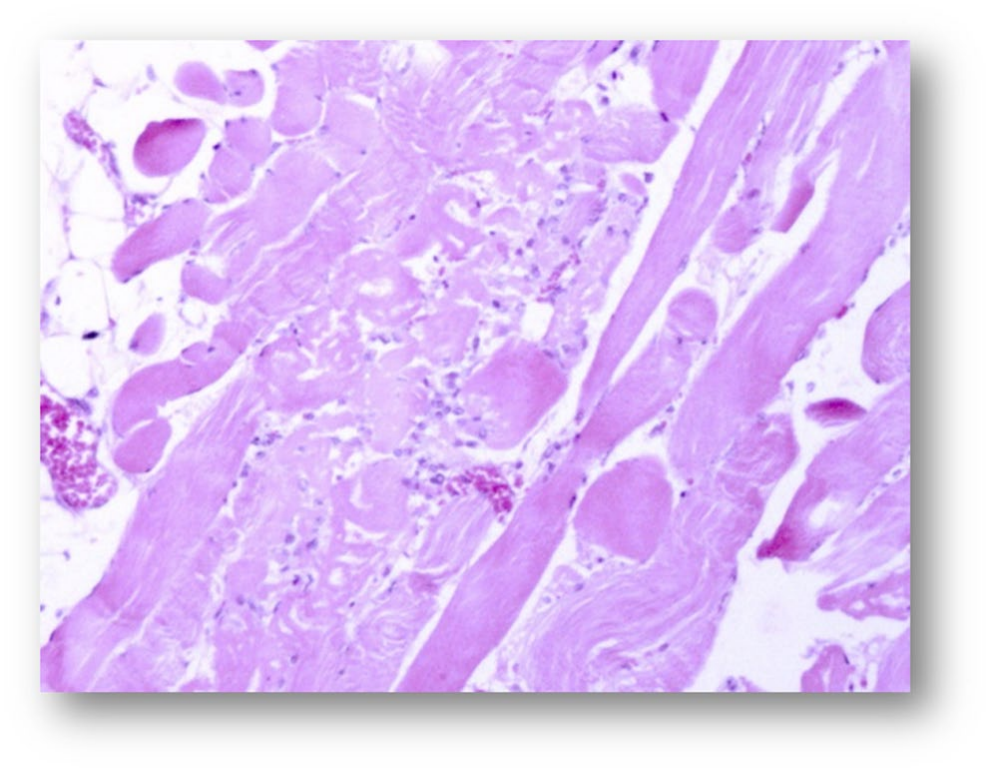
Pathohistological micrograph of the skeletal muscle from animal 2 (magnification ×10) after haematoxylin and eosin staining

**FIGURE 6:**
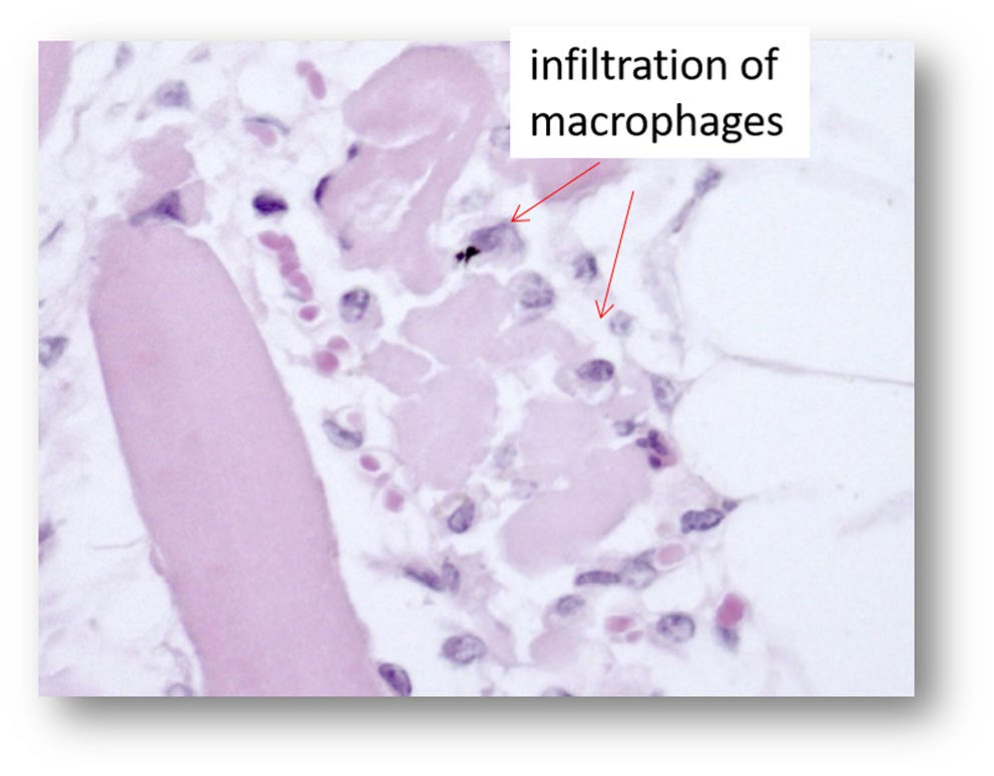
Pathohistological micrograph of the skeletal muscle from animal 1 (magnification ×40) after haematoxylin and eosin staining

The skeletal muscles showed a moderate to severe acute monophasic myonecrosis (rhabdomyolysis) with loss of the cross striation in both examined animals (Figures 3, 4, 5, 6). The samples from the M2 milu, which died 1 day later, showed a mild infiltration of neutrophils and the presence of macrophages (Figure 6). The myocardium of both animals showed only mild changes, including oedema, loss of cross striation, or necrosis of single myocytes (not shown).

### 3.4 Detected concentrations in serum/urine and organ/digesta samples

Blood sample analysis showed greater CK activity (U/L: M1 312,000; M2 338,000) in comparison to a reference from cattles (<150), and the same was observed with LDH (U/L: M1 16,000; M2 15,000, ref <150; Moritz, 2014). This finding indicated severe muscle cell damage. The detected amounts of HGA were high for M2 (480 nmol/L) and M3 (460 nmol/L), but MCPrG was below the limit of detection (Table 1). As there were no comparative data from milus or other ruminants, we used data from horses for comparison.

**TABLE 1:** HGA, MCPrG, their specific toxic metabolites (MCPA‐G/MCPA‐C and MCPF‐G/MCPF‐C), and selected acyl carnitines in serum and urine samples of affected Pére David's Deers compared to data from equines

Item	Serum (nmol/L)		Urine (nmol/mmol creatinine)		Control^a^ (horses)
	Milu 2	Milu 3	Milu 2	Milu 3	
HGA	480	460	5	4	<dl
MCPA‐G	740	190	16,800	4,600	<dl
MCPA‐C	500	140	680	330	<dl
MCPrG	1	<dl	<dl	<dl	<dl
MCPF‐G	940	110	7,500	1,800	<dl
MCPF‐C	18,300	9,600	21,300	14,000	<dl
**Item**	**Serum (µmol/L)**		**Urine (µmol/mmol creatinine)**		
Butyryl‐C	290	170	250	190	<0.2
Isobutyryl‐C	50	30	140	80	<0.5

Abbreviations: dl, detection limit <1 nmol/L; HGA, hypoglycin A; MCPA‐C, methylenecyclopropylacetyl‐carnitine; MCPA‐G, methylenecyclopropylacetyl‐glycine; MCPF‐C, methylenecyclopropylformyl‐carnitine; MCPF‐G, methylenecyclopropylformyl‐glycine; MCPrG, methylenecyclopropyglycine.

^a^ Data from horses were used for comparison with concentrations in serum and urine (Bochnia et al., 2019; Bochnia et al., 2015).

Concentrations of both toxins were high in the rumen contents, the liver of M3, and the faeces of M2 and M3, but not in the kidney of either milu (Table 2). The toxic metabolites of HGA and MCPrG were high in the serum, urine (Table 1), liver and also partly in the kidney, but not in the faeces (Table 2). The detected concentrations of butyryl‐C and isobutyryl‐C were increased in the serum (Table 1), but this was not identified in the methanol extracts of the tissue samples (Table 2).

**TABLE 2:** HGA, MCPrG, their specific toxic metabolites (MCPA‐G/MCPA‐C and MCPF‐G/MCPF‐C) as well as selected acyl carnitines in organ samples, digesta and faeces of affected Pére David's Deers compared to data from equines

Item	Unit	Rumen digesta		Liver		Kidney		Faeces		Control^a^
		Milu 2	Milu 3	Milu 2	Milu 3	Milu 2	Milu 3	Milu 2	Milu 3	
HGA	nmol/L	1,700	1,800	<dl	<dl	<dl	<dl	300	2,000	<dl
MCPA‐G	nmol/L	3	10	3,600	1,100	1,100	270	5	10	<dl
MCPA‐C	nmol/L	<dl	<dl	20	20	10	8	<dl	<dl	<dl
MCPrG	nmol/L	920	860	2	1,200	1	6	9	1,400	<dl
MCPF‐G	nmol/L	4	2	700	240	240	80	4	1	<dl
MCPF‐C	nmol/L	3	5	4,700	1,500	590	200	3	3	<dl
Butyryl‐C	µmol/L	<dl	<dl	50	30	9	6	<dl	<dl	<0.2
Isobutyryl‐C	µmol/L	<dl	<dl	8	5	3	2	<dl	<dl	<0.5

Abbreviations: dl, detection limit <1 nmol/L; HGA, hypoglycin A; MCPA‐C, methylenecyclopropylaceticacid‐carnitine; MCPA‐G, methylenecyclopropylaceticacid‐glycine; MCPF‐C, methylenecyclopropylformyl‐carnitine; MCPF‐G, methylenecyclopropylformyl‐glycine; MCPrG, methylenecyclopropyglycine.

^a^ Data from horses were used for comparison (Bochnia et al., 2019; Bochnia et al., 2015).

## 4 DISCUSSION

This study has confirmed that, in addition to HGA, the second homologous toxin MCPrG is involved in AM in milus. Similar to Bunert et al. (2018), the proximity of SM trees to the enclosure of the milus and, in this study, feeding them the plant residuals after pruning led to an exceedingly severe course of the disease and three dead milus within less than two days. No case of AM had been previously observed at the Zoo in Dresden. The seeds and the leaves of the SM tree contained very high concentrations of both toxins, within the well‐recognized high variation observed in previous studies (Baise et al., 2016; Bochnia et al., 2015, 2019; Żuraw et al., 2016). It appears that it is not only the quantities of the ingested plant material, but also the leaves and seeds individually selected by the animal that impact the ingested doses of the toxins and determine whether the effects are toxic or not. It has been previously shown that very small quantities of highly toxic seeds/samaras are able to poison a 500 kg horse (Baise et al., 2016; Valberg et al., 2013). There is evidence that pruning and therefore the stress level of the cut plant increases the levels of HGA and MCPrG in plant materials (e.g. seedlings), and therefore the ingested amount of the toxic compounds. In previous studies, the HGA content in young seedlings was significantly higher after mowing, especially within the first 2 days after cutting (González‐Medina et al., 2019). In this case, the plants are able to produce toxic substances that have deterrent or toxic effects (e.g. in animals; Hulme & Benkman, 2002; Laycock, 1978), which may also apply to the toxic amino acids.

The milus showed the typical clinical symptoms of AM and a rapid progression in the disease, similar to horses (Bochnia et al., 2015; Valberg et al., 2013; Żuraw et al., 2016) but also similar to milus (Bunert et al., 2018) with trembling, recumbency, depression, weakness and myoglobinuria; in such cases, the animal is often unable to urinate because of paralysis of the bladder (Bochnia et al., 2015). Therefore, urination often occurs after death, as we observed in this study (Figure 2).

The pathological findings in the milus such as the signs of a severe, acute and monophasic rhabdomyolysis, and the loss of the typical cross striation were similar to the results from previous studies with milus (Bunert et al., 2018) and horses (Żuraw et al., 2016). The high levels of creatinine kinase and lactate dehydrogenases measured in the serum were indicators of muscle cell damage; such elevated levels have been described previously in horses (Bochnia et al., 2015, 2019; Żuraw et al., 2016) and milus (Bunert et al., 2018), with concentrations >300,000 U/L for creatine kinase, which is 3,000‐fold higher than the reference range for cattles (Moritz, 2014).

The accumulation of both of the selected acyl conjugates (butyryl‐C and isobutyryl‐C) in the blood and urine, which mainly appearing during the metabolism of MCPrG via MCPF‐CoA, demonstrated on the one hand the well‐known inhibition of the β‐oxidation of fatty acids associated with AM, but on the other hand the involvement of MCPrG (Bochnia et al., 2019; Sander et al., 2019). A high correlation between both acyl‐carnitines and the existence of MCPrG during intoxication has been demonstrated in horses previously (Bochnia et al., 2019). Moreover, the involvement of MCPrG is supported by the detection of the specific metabolites in the serum and urine, particularly the very high concentrations of MCPF‐C in both body fluids (serum and urine) being 40‐fold higher than MCPA‐C. Levels of MCPF‐G and MCPA‐G were also high in the urine. In general, urine appears to be the preferable matrix for the detection of glycine derivatives, because the specific metabolites MCPA‐G and MCPF‐G were 10–20‐fold higher in urine than in serum. As was shown in previous studies (Bochnia et al., 2019; Sander et al., 2019) HGA but not MCPrG (isomer 1 and 2) was mainly identified in the serum samples. Sander et al. (2019) described two isomers of MCPrG, whereas Isenberg et al. (2016) and Sanford et al. (2018) did not differentiate isomers during the analytical process. Both isomers appear to have different specifities for enoyl‐CoA‐hydratases 1 and 2 during the second step of β‐oxidation, which may affect the degrees of inhibition in the peroxisomes or mitochondria (Li et al., 1999; Wu et al., 2008). Therefore, further research is needed to elucidate the additive toxicological effects of HGA and MCPrG, and also to better understand the role of the specific isomers of MCPrG. A possible explanation for the absence of MCPrG in the serum may be its particularly fast metabolism, because MCPrG was found at similarly high levels to HGA in the rumen digesta. None of the known toxic metabolites were produced and detected in rumen digesta, which substantiates the hypothesis that HGA and MCPrG are mainly metabolized after absorption rather than by microbial fermentation in the intestinal tract (Krägeloh et al., 2017). However, we do not know whether microbial fermentation of the toxins leads to other metabolites. An outbreak of AM might thus depend on the absorbed amounts of the toxic AAs which are transported via the bloodstream to the liver (Krägeloh et al., 2017).

No cases of AM were reported in ruminants until a retrospective study was published in 2018 (Bunert et al., 2018) showing that ruminants are also at high risk of becoming ill (25/77 deers, approximately 33% of all deers in Duisburg Zoo) if they ingest substantial quantities of seeds or seedlings from SM trees. Protection by ruminal microorganisms against toxic AA cannot be confirmed currently.

High concentrations of metabolites from HGA and MCPrG (MCPA‐G, MCPF‐G and MCPF‐C) were found in the liver and kidney. HGA and MCPrG were detected in some rumen and faeces extracts, and MCPrG was also detected in the liver of the affected milus. Two links to the food chain should be briefly addressed here: (a) there is a question of whether the consumption of offal, such as the liver or kidneys, but also meat and other products from affected wild or domestic ruminants by humans is safe, and this remains unclear at the moment; (b) a possible transfer of HGA into milk or at least a diaplacentar transfer was shown in a lactating mare affected by AM, and subsequent intake by the foal led to intoxication and sudden death within a few hours after birth (Karlíková et al., 2018). Therefore, intoxication may also occur, or at least cannot be excluded, from products of lactating animals used for human consumption. A high variation in the amount of HGA and MCPrG, as well as the ratio of both, in the investigated plant materials, influences the ingested doses. Variable concentrations can be affected by the ripeness of SM seeds or seedlings, or the vegetation period, which has been shown for distinct *Sapindaceae* fruits (Bowen‐Forbes & Minott, 2011; Li et al., 1999). However, the absence of MCPrG in the serum combined with distinctly higher concentrations of the specific metabolites in the liver, kidney and serum as well as urine substantiate the presumption that the metabolism of MCPrG is faster than for HGA. This implies that MCPrG might on the one hand accelerate and increase the toxic effects in AM, which has been shown previously in horses (Bochnia et al., 2019) and has now been confirmed in ruminants through this study. On the other hand, it could suggest that MCPrG has a higher impact on the course or the pathophysiology of AM as suspected by Bochnia et al. (2019) and Sander et al. (2019), which is supported by the higher concentrations of the specific toxic metabolites.

## 5 CONCLUSIONS

Our results show that MCPrG is involved in AM in Pére David's Deers, otherwise known as milus. The low MCPrG concentration in serum may indicate that MCPrG is metabolized faster than HGA and therefore specific metabolites appear highly concentrated, in the form of derivates. HGA and MCPrG represent a major hazard for zoo animals, as they do for equines and humans. Similar to non‐ruminants (monogastric animals such as humans and equines), metabolism of the toxic AAs (HGA and MCPrG) by microbes in the digestive tract, if any, or by the environment does not seem to protect against their absorption. The possibility of human intoxication, via the food chain, resulting from the consumption of products from affected ruminants should be taken into consideration.

## CONFLICT OF INTEREST

The authors declare that they have no conflicts of interest. None of the authors have financial or personal relationships that could inappropriately influence or bias the content of the paper.

## AUTHOR CONTRIBUTION

**Mandy Bochnia:** Conceptualization; Data curation; Formal analysis; Investigation; Project administration; Supervision; Visualization; Writing‐original draft; Writing‐review & editing. **Eva Ziemssen:** Data curation; Investigation; Resources; Writing‐review & editing. **Johannes Sander:** Formal analysis; Investigation; Methodology; Resources; Validation; Writing‐review & editing. **Birgit Stief:** Data curation; Investigation; Methodology; Resources; Writing‐review & editing. **Annette Zeyner:** Conceptualization; Project administration; Supervision; Writing‐review & editing.

### Peer Review

The peer review history for this article is available at https://publons.com/publon/10.1002/vms3.406.
